# Some Ideas on the Treatment and Management of Typhoid Fever, and Other Diseases, in Ante-Bellum Days

**Published:** 1912-06

**Authors:** 


For Texas Medical Journal.

### Some Ideas on the Treatment and Management of Typhoid Fever, and Other Diseases, in Ante-Bellum Days.

BY A RETIRED TEXAS PHYSICIAN.

Time was, when, as they now frequently are, the typhoid patient was kept in rooms, shut out from drafts of fresh air. Even as early, or possibly earlier, than 1859, the physician whom

I have previously mentioned, Dr. F. L. Merriwether, nearly always put his typhoid patient out on a piazza, and sometimes, in summer time, put his bed out under the shade of a big tree, where the general wave of fresh air could sweep over him, because the cooler atmosphere would moderate the temperature of his body. A patient, in the spring, summer and fall time burning with fever, as we sometimes express it, has a hot time of it in a room where the breeze does not strike him. Suppose the temperature has risen to 105° F., and he is restless and moaning, has *subsultus tendinum*, picking at the bed clothing, etc., he is not likely to have a modification of that condition very soon, if at all. Now then, if a breeze is blowing, wheel his bed across a large open window, where the wind can strike him squarely, with only a shirt to cover him, and a sheet alone thrown across the lower extremities. Leave him to the nurse, for two hours, then return, and take his temperature, and if his temperature has not fallen one, or even two degrees, and the symptoms mentioned have not considerably improved, you may "spit in my face and call me a horse." But if you put him on the piazza, or out doors, where the breeze is general, you will find, frequently, and almost surely, that the improvement is still greater, for the patient is very apt to be sleeping quietly, much to your satisfaction. Rest assured that a modification of these symptoms, by so simple a remedy, though the modification may be only temporary, always helps to preserve his strength, and helps to prevent bad symptoms from growing worse. The strength of the breeze whips the cooler temperature of the atmosphere into and nearly through his very skin. It serves as the better and more conservative method of lowering the temperature than that of the fatiguing method of lifting him into a bath tub and lifting him out again, for he may be kept in the open air indefinitely. The latter is rarely, if ever, a very satisfactory procedure. It may do, if there is no other way possible. As a matter of experiment, I have frequently closed up the windows and doors of a fever patient's room, took his temperature, and after an hour or two I would return, immediately open up everything and put the bed across a window opening. The temperature would drop to former condition. My nurses, upon observing the marked difference, learned something, and afterwards kept everything open, as I commanded.

As for medical treatment, I have nothing to say, for we are all, even now, up in the air.

ACUTE DELIRIUM TREMENS.

CASE No. 1.—In Houston county in 1859 and 1860, a man of about 35 years of age, a merchant, would get on a spree occasionally. This time, however, he had been steadily drinking for about two weeks, lost desire for food, and had eaten but little the last few days. Was called and found him as wild as a "March hare," and two negro men holding him in bed. This was in 1860, while I was beginning to practice, assisting my old preceptor. The patient was, by accident, at a farm (plantation) house owned by a friend. I was up against my first case of the kind, but I recollected the reading of an account, in an old newspaper, where a Dr. Carpenter, I believe, had suggested the removing the cause by sweating, but said nothing of the method. I nerved myself for the task by having a large, old fashion outdoor boiler pot filled with water, and set to boiling, with fire under it, of course. Then, as I directed, the negroes dipped thick woolen blankets into it, wrung them so they would not drip too freely, and in great folds laid them on his body—not too hot, of course—and on his extremities, while he was lying on a sheet and a wet blanket on the floor. A *cold cloth* was kept on his forehead all the time. I began this process about sundown, and kept it up until morning, changing blankets occasionally, when they began to get too cool. He sweated profusely, and was wild until about 1 o'clock, when a gradual change commenced. He grew less restless, and at 4 o'clock he went to sleep, and slept soundly until about 9 o'clock next morning. He awoke, and was astonished at his surroundings, and at the sight of me. "Why, doctor, how is this, and what are *we* doing here?" His mind was nearly sound again, and he ate some breakfast, but as he seemed to be a little off every now and then, I explained it all, and he allowed me to sweat him two or three hours longer. He ate heartily at 1 o'clock, and then said he felt that he was all right. At 6 p. m. he was sitting up. The next morning he rode home, a distance of eight miles, feeling all right again.

When I first walked into his room he was terribly frightened, exclaiming: "Look there, the doctor is going to shoot me, and he never misses." "D——n these horns; how can I get them through the bushes before he gets near me." He and the old doctor had frequently hunted deer with hounds, and the patient imagined that he himself was a big buck with great horns, and didn't know how to get them through the brush. I was proud of my first great success. Why is it that many, even in this day, do not use this great eliminative treatment to remove the cause? I do not remember having seen any suggestion of this treatment.

*Opium.*—Has anyone ever used this eliminative process for opium eaters—persisting in the treatment for a longer time, rather than the painful cures now in vogue? Somehow I have never had an opportunity to try it.

After this sweating treatment, which eliminates the alcohol, the odor of it is discovered when the patient's bed is uncovered. Then the patient is restored to a normal condition, and, *strange to say, has* no desire whatever for alcohol, for a long time, possibly altogether; because his system has assumed normal function, being free from exciting cause.

CASE NO. 2.—A merchant, in C., who made his own saddles, bridles and harness, and I sold them, took to hard drink. He had heard me tell of the first case, so one evening (afternoon as they now say) he sent for me, and said to me, in an excited way: "I am getting into a h—ll of a fix. I know it from—see that d——d monkey now on the roof—what you said, and I want relief—there he goes now, d——n him—before it is too late." I prepared as before, with the pot of boiling water, two negro men to sit up and do the work. In his case, after the first four hours he slept and sweated until morning, leaving his mind clear as a bell, ready for a good breakfast. I showed him his bed sheets, and he could smell the whisky.

I have had other cases of this kind. These are simply two typical cases.

These cases are fine illustrations of the removal of the cause by elimination. The cure was effected by simply removing the cause.

"Wet heating" as I style it—by blankets wrung out of hot water—serves a double purpose. This plan not only sweats the patient and removes the cause, but during the process it aids very materially by acting as a nerve sedative—and there is no better in certain conditions—helping, by its soothing effects upon the nervous system, to quiet the excessive delirium.

NICOTINE ELIMINATION.

CASE NO. 3.—This case was, as I supposed from the history of the case, caused by nicotine poisoning, and upon that supposition I cured him by elimination. He dropped into my drug store, and told me how bad he felt, and that he was uneasy about himself. His memory was sadly defective; he could not sleep much, because he was nervous; had an aversion during the last week for food; felt weak, and thought his heart was not exactly right. "Feel my pulse," said he, "it sometimes beats entirely too slow, and see I have fallen off thirty pounds in three months." As he was an intimate friend, I knew his habits, and I went at him at once. I suppose he was nearly 50 years of age. Said I, "Young man, I will be plain with you. You are an inveterate and persistent user of tobacco. Give me that pipe, and while you are about it let me have your tobacco." I threw both out into the gutter. "Now, sir, you must positively quit this habit, for you are badly poisoned with nicotine, a dangerous alkaloid of tobacco, and are liable to drop off just at any moment. Your life is just clinging to the ragged edge of nothing, and common sense dictates that you do as I say, if you desire to live." My earnestness alarmed him, and he promised to follow my advice. Said I, "Go home at once, take a warm bath, and then sweat yourself for an hour or so with blankets wrung out of hot water, then pour a pitcher of moderately cold water over your head and shoulders, and wipe off with a coarse towel. Do this twice every day and recline on a bed or couch during intervals. Be sure to drink sweetmilk occasionally. Keep up this treatment for seven days, then report to me." The treatment was so simple that he said: "Is that all?" "Yes until you report again, for I must sweat the poison out the first thing—then come." "Oh," said he, "I see." He did as I told him, but on the fifth day he came in looking fine, and said: "No use sweating any more, I am all right now, and want to eat five meals a day. Anything else?" "No," said I, "but you must let tobacco alone." "Why," said he, "I have no *desire* whatever for it now." The cause had been removed.

CASE No. 4.—Some 18 or 20 years ago I went to the San Antonio hot sulphur well—three miles out—for stomach trouble; found that it was of no benefit to me, but while there I met a district judge whom I knew by reputation, a lawyer of much ability. I knew many public men of his acquaintance in this State. He was not well; had lately become so nervous that he did not sleep, or eat well; memory was failing. It was difficult to be comfortable on the bench, and was becoming quite feeble; heart not right, etc. This is my recollection of his symptoms. I noticed that from "early morn till dewy eve" an old pipe was never out of his mouth. Said I: "Judge, will you allow me to venture a suggestion concerning your case?" "Certainly," said he, "for I

am here merely to try the hot baths, and drink the water, through the suggestion of some friend." "I believe, as you have been an inveterate smoker so long a time, that you are poisoned with nicotine, a poisonous alkaloid of tobacco, and you can be cured right here, without medicine." He had been in the habit, simply, of taking a hot bath and a glass of hot well water, then he would rest for an hour on a lounge, in the ante-chamber, before taking a walk, for he was too much debilitated to go out at once from the bath. Now notice the difference—owing to the way you do things. I instructed the bath attendant to let him stay in the bath fifteen minutes, then lay him on a couch, sweat him with heavy hot water blankets for one hour, then stand him up, and pour a large pitcher, or good-sized bucketful, of moderately cold water all over his head, shoulders, arms and body, and wipe him dry with a rough towel. I waited for him the first time after the bath and sweating, to see how he liked it. Directly he came out, and slapped me on the shoulder. "Doctor," said he "I am ready for the walk now, for that sweating, followed by the cold douche, invigorates me." After three days more of sweating and douches twice a day, he went away to San Antonio, feeling all right again, he said, but he threw away the old pipe, his enemy and his companion for many long years. He could do so easily, for, as in nearly all cases of complete elimination, the *desire* was gone. How different is this from the so-called cures which are effected, if at all, by counter-effect or counter-action through some medicinal agent, often leaving the desire simply in abatement. Elimination absolutely, in poisoning by alcohol, nicotine, morphine, etc., removes the cause of the trouble, and, consequently, the desire, and does it promptly.

These four cases are suggestive. If we can eliminate whisky, and nicotine, why not try to cure the morphine habit by the same treatment? You know that—from what morphine patients tell us—that, when they attempt to leave off the usual doses, distressing symptoms appear, either of pain or discharges from the bowels, etc. Now if they were treated, as I treated the cases of delirium tremens, steadily for from fifteen to twenty hours, repeating this process, if necessary, after a few hours of rest, I believe that while the quantity is being rapidly lessened by elimination the hot soothing effects of the *moist*-heat (steam) would prevent the distressing symptoms. When the morphine was once out, the desire would be gone, and normal conditions would soon build up the patient. I suggested it once to a physician, who said he was curing patients with his "opium habit cure," but he was satisfied with his method, and would not try my idea. It was too simple, perhaps.

Some one should try it in his sanitarium, if he has not tried it already.

The methods of sweating, in this day, by pilocarpin, the sweat box, etc., will hardly substitute for the wet heat by blankets, etc.

